# Manual flagging failed to identify pseudohyperkalemia in acute myeloid leukemia: case report

**DOI:** 10.1186/s12245-024-00734-x

**Published:** 2024-10-09

**Authors:** Yangming Cao

**Affiliations:** 1 Division of Nephrology, Department of Internal Medicine, UCSF Fresno Center for Medical Education and Research, 155 N Fresno St, Fresno, CA 93701 USA; 2 The Nephrology Group, 568 E Herndon Ave, Suite 201, Fresno, CA 93720 USA

**Keywords:** Pseudohyperkalemia, Acute myeloid leukemia, Pneumatic tube transport, Lab flagging, Automation, Case report

## Abstract

**Background:**

Pseudohyperkalemia is well known in acute or chronic lymphocytic leukemia, but it is very rare in acute myeloid leukemia (AML). The lab flagging system for leukocytosis to prevent pseudohyperkalemia may not work.

**Case presentation:**

A 55 year-old white man with AML was sent to emergency department for transfusion due to severe anemia. Blood test showed severe leukocytosis and elevated potassium. Repeated blood test showed his potassium was even higher. Anti-hyperkalemic medical treatment was given. He was then diagnosed with pseudohyperkalema.

**Investigation:**

I was repeatedly reassured that the lab’s manual flagging system for leukocytosis was the key in reaching the correct diagnosis. My persistent inquiries, however, revealed that the flagging system was not functioning in the care of this patient. It was clinicians’ suspicion of pseudohyperkalema that led to the correct diagnosis, although the clinicians’ recommendation of obtaining a heparinized plasma for test did not play a role because all blood samples were already heparinized. The cause of pseudohyperkalemia was pneumatic tube transport. After this incident, our laboratory is investigating the options of using the Laboratory Information System to automatically flag the results and Clinical Laboratory Scientists to make the chemistry team more aware of potentially erroneous potassium results due to pseudohyperkalemia.

**Conclusions:**

Pseudohyperkalemia associated with leukocytosis still occurs. This is the first case of pneumatic tube transport causing pseudohyperkalemia associated with AML. When significant leukocytosis, thrombocytosis, hyperproteinemia, or hyperlipidemia is present, whole blood should be utilized for potassium measurements and walked to the lab instead of sent by pneumatic tube transport. Even in a lab with a manual flagging system, there is still room to improve by implementing an automatic flagging system.

## Abstract (pa)

Despite an existing laboratory flagging system, pseudohyperkalemia still occurred in a patient with extreme leukocytosis. The incident led to a plan to automate the flagging.

## Background

Pseudohyperkalemia has been widely reported in acute or chronic lymphocytic leukemia [1, 2]. In contrast, it is very rare in acute myeloid leukemia (AML) [3–6]. Here I reported the first case of pneumatic tube transport (PTT) causing pseudohyperkalemia in acute myeloid leukemia (AML). Despite the manual flagging system for leukocytosis that initially appeared to work, a persistent investigation revealed that the flagging was not functioning in this patient’s care. The process of investigation and our plan to improve the flagging system were described.

## Case presentation

A 55 year-old white man was sent to emergency department (ED) for transfusion on April 3, 2024. He was diagnosed with multiple myeloma in 2016 which, despite multimodality treatment, transformed to myelodysplastic syndrome in 2022, culminating in acute myeloid leukemia (AML) in March 2023. He underwent combination chemotherapy for AML until January 2024. In late March, 2024, a bone marrow biopsy showed worsening AML with 80% myeloblasts by CD34 in the core and 70% blasts in peripheral blood. Hydroxyurea was started, but he tentatively chose not to be admitted for further chemotherapy. On April 2, 2024, he received potassium chloride 40 mEq intravenously (*i.v*.) as an outpatient for low plasma potassium (K^+^) at 2.8 mEq/L. He had been on fludrocortisone and hydrocortisone for adrenal insufficiency that developed in 2019 and also on oral potassium chloride (20 mEq p.o. bid) for chronic hypokalemia that developed in May 2023.

Then on April 3, 2024, a blood test (Sample A, Table 1), drawn at his home by a home health nurse and sent to our hospital lab via courier service and walk, showed a hemoglobin of 4.5 g/dL (compared with 7.5 g/dL two days prior) and K^+^ of 3.0 mEq/L. Immediately he was referred to our ED for transfusion. Exam revealed normal vital signs without acute distress.

**Table 1:** Blood sample handling and test methods of our patient

Time drawn	4/3/2024 12:00 pm	4/3/2024 20:03 pm	4/3/2024 20:48 pm	4/3/2024 23:37 pm
Sample	A	B	C	D
Tests	Met 15	Met 10	Met 10	Met 10
Potassium(mEq/L)	3.0	6.7	7.8 ^A^	3.6 ^B^
Setting	Home	ED	ED	ED
Collection tube^C^	Light green top	Dark green top	Dark green top	Dark green top
Anticoagulant ^D^	Lithium Heparin	Lithium Heparin	Lithium Heparin	Lithium Heparin
Sample transport	Courier and Walk	Pneumatic tube	Pneumatic tube	Walk
Blood tested	Plasma	Whole blood^E^	Whole blood^E^	Whole blood^E^
Lab location	Main lab	Main lab	Main lab	Main lab
ChemistryAnalyzer	Beckman Coulter AU5800	NovaBiomedical Stat Profile pHOx Ultra	NovaBiomedical Stat Profile pHOx Ultra	NovaBiomedical Stat Profile pHOx Ultra

^A^ After Potassium of 7.8 mEq/L was reported, anti-hyperkalemic medical treatment was administered (see text); ^B^ Obtained about 30 min after anti-hyperkalemic medical treatment; ^C^ BD vacutainer blood collection tube; ^D^ Coating inside of collection tube; ^E^ Without centrifugation

In ED, blood test showed white blood cells (WBC) of 206.8 × 10^3^/µL with 90% blasts, hemoglobin of 7.9 g/dL and platelets of 38 × 10^3^/µL. Obviously hemoglobin of 4.5 g/dL was a lab error. His Met 10 panel (Sample B, Table 1) showed K^+^ 6.7 mEq/L. The ED physician requested a redraw of blood (Sample C, Table 1) revealing K^+^ 7.8 mEq/L. Hemolysis was ruled out by observing the supernatant of centrifuged blood after the chemistry blood test was completed. Other blood tests included phosphorus 2.6 mg/dL, ionized calcium 5.0 mg/dL, uric acid 4.7 mg/dL, glucose 193 mg/dL, BUN 9 mg/dL and creatinine 1.2 mg/dL. Despite that he had no associated symptoms and his EKG showed no hyperkalemic changes, anti-hyperkalemic treatment with calcium gluconate (*i.v*.), sodium bicarbonate (*i.v*.), regular insulin with 50% dextrose (*i.v*.) and albuterol nebulizer, was immediately administered. Nephrology consult was called for possible need of hemodialysis. The nephrologist on-call rightly suspected pseudohyperkalemia and recommended obtaining a heparinized plasma sample to re-test. The ED physician relayed the request to the Chemistry lab team who addressed the issue appropriately, and a repeat blood K^+^ came back at 3.6 mEq/L (Sample D, Table 1). Pseudohyperkalemia was thus ascertained, although the recommendation of obtaining heparinized plasma was irrelevant because his blood was already heparinized (Table 1). Of note, Sample D was obtained about 30 min after started on anti-hyperkalemic treatment. After the K^+^ was shown to be normal, one unit of packed red blood cells was transfused. Choosing comfort care, he was discharged home with stable vital signs the next day (April 4, 2024) and died one week later.

## Investigation

The next day (April 4, 2024), I took over the nephrology service and started to investigate the process that had led to the correct diagnosis, with a goal of preventing similar incidents. When I entered the main lab of the hospital, most of the lab staff appeared ready to answer my inquiry, although the lab technicians / clinical lab scientists ((LT/CLS) working the previous night were not present. Two lab LT/CLS independently told me the followings: (a) there is a small lab in the ED for some basic tests including chemistry tests (using the same machine and the same heparinized whole blood for test as in the main lab); (b) but that night, the ED lab was closed due to staff shortage, and so all ED blood samples (the heparinized whole blood in our case) were sent to the main lab via PTT which was the cause of the problem; (c) we have a flagging system: once Hematology LT/CLS see high WBC (> 100 × 10^3^/µL), they immediately alert Chemistry LT/CLS who, in turn, tell phlebotomists to redraw using heparinized whole blood and walk the blood sample to the lab (i.e., not by PTT); (d) then the Chemistry LT/CLS do the test, resulting in normal K^+^ level. They both reassured me that it was our flagging system that solved the problem, namely, the lab flagging worked before clinicians’ reminder. Interestingly, if the ED lab was open that night, the blood sample would be walked to the ED lab and pseudohyperkalemia would not have occurred. That reminds us that even in the same hospital on the same day, pseudohyperkalemia can suddenly happen because of different handlings of blood samples. By the way, what was tested in Sample A was heparinized plasma instead of heparinized whole blood (Table 1).

But I still wondered why there was a redraw (Sample C) if the flagging system was working. The high K^+^ from Sample B together with the flagging of leukocytosis from Hematology LT/CLS should have made Chemistry LT/CLS take appropriate actions immediately. Then pseudohyperkalemia would be readily identified, obviating the need of therapy and nephrology consultation. One month later I went to the lab again, and two other lab LT/CLS gave me the same answer as before. A few days later, I asked the lab supervisor to talk to the LT/CLS who worked that night. Three days later, he told me that the Hematology LT/CLS did not inform Chemistry LT/CLS, although all lab staff (including Hematology team and Chemistry team) worked in the same large room. Only after clinicians’ reminder of possible pseudohyperkalemia did the Chemistry LT/CLS take the appropriate steps, solving the problem. The main finding was that our manual flagging system requires Hematology LT/CLS to make Chemistry LT/CLS and phlebotomists aware of potential issue with elevated WBC counts. We also found that only a Technical Specialist (a very senior CLS who has worked as CLS for more than 15 years) in Chemistry can add the flagging comment in middleware (which is viewable only to lab staff). Technical Specialists are not on shift 24/7 to add the comment, creating another opportunity to miss these specimens. After this incident, our laboratory is investigating the options of using the Laboratory Information System to automatically flag the results and CLS to make the chemistry team more aware of potentially erroneous K^+^ results due to pseudohyperkalemia.

## Discussion and conclusions

Since spurious hyperkalemia was first described by Hartmann and Mellinkoff in 1955, there have been abundant case reports of pseudohyperkalemia (falsely elevated potassium in serum) and reverse pseudohyperkalemia (falsely elevated potassium in plasma) [1, 2]. Avelar compiled a relatively comprehensive list of causes for pseudohyperkalemia [7]. All of these same factors such as mechanical stress also apply to reverse pseudohyperkalemia [2, 7]. Pseudohyperkalemia is mostly associated with severe thrombocytosis or leukocytosis as in acute or chronic lymphocytic leukemia, with one of the main mechanisms being in vitro potassium release into serum from cells during clotting formation [1, 2]. Another theory suggests that severe leukocytosis has higher consumption of metabolic fuels that may lead to impaired Na^+^/K^+^ ATPase pump activity, which leads to potassium release from the high number of WBCs [2]. Heparin-mediated cell membrane damage during processing and centrifugation in the context of hematologic malignancy is a risk for reverse pseudohyperkalemia [7]. In contrast, the formation of a fibrin clot in serum specimens is hypothesized to entrap and stabilize tumor cells during centrifugation, avoiding pseudohyperkalemia [8].

Pneumatic tube transport (PTT) system was famously introduced to Mayo Clinic by Dr. Henry Plummer in 1928 and now is being used by many hospitals around the world [9]. For example, our hospital’s PTT, delivering about 4,000 packages a day, can reach a linear speed of 17 mph with rapid accelerations and decelerations, as well as angular velocity changes with turns. Pneumatic transport of specimens with normal WBC counts did not affect the potassium measurement [10, 11]. However, Kellerman, et al., described the first case of pseudohyperkalemia caused by pneumatic tube transport of blood specimens from a patient with extreme leukocytosis [11]. The patient apparently had acute lymphocytic leukemia transformation originating from his prior mantle-cell non-Hodgkin lymphoma. The different effect of PTT on specimen with normal WBC counts vs. extreme leukocytosis was attributed to both high WBC number and cellular fragility of malignant cells. His study effectively excluded other factors such as vacutainer draw from a venous catheter, venous sample draw, lithium heparin (vs. heparin alone) as a cause of pseudohyperkalemia. In essence, Kellerman’s and our case were both reverse pseudohyperkalemia, although no serum sample was tested.

In a striking comparison to lymphocytic leukemia (acute or chronic), AML has been rarely associated with pseudohyperkalemia. This may be due to different cellular fragility between lymphocytic leukemia cells and AML blasts. There are only four reported cases, published in English, on pseudohyperkalemia related to AML (3–6 see Table 2). One of the cases was published in French with an English abstract. An attempt to reach the author by email for case details was unsuccessful [5]. The falsely elevated serum potassium level in all of the other 3 cases was due to blood clotting. Our patient was the first case of PTT causing pseudohyperkalemia in AML. The reduction of K^+^ by the medical treatment was expected to be at most 1.80 mEq/L in our patient (0.90 mEq/L each for insulin and albuterol, respectively, while no change was expected with sodium bicarbonate) [12]. Thus the plasma K^+^ would, at best, be lowered from 7.8 to 6.0 mEq/L which was still much higher than 3.6 mEq/L (the real K^+^ level of our patient). Therefore there was certainly a significant component of pseudohypokalemia. Clotting did not play a role because all blood samples were heparinized.

**Table 2:** Case reports of pseudohyperkalemia in AML

Authors Year (Ref.)	Chumbley 1970 [3]	Salomon 1974 [4]	Goubella 2013 [5]	Darapu 2014 [6]	Our patient 2024
Age (years)	68	39	Unknown	71	55
Race/Gender	White/Male	Female	Unknown	Male	White/Male
WBC (x10^3^/µL) Blast type Blast percent	285 Meyeloblast 89.5%	162 Meyelo-monoblast 79%	Unknown Unknown Unknown	185 AML-M7 Unknown	206 AML CD34 90%
Serum K^+^mEq/L^A^ PlasmaK^+^mEq/L^A^ Whole blood K^+^	7.7 4.6 Not tested	7.5 3.2 Not tested	Unknown Unknown Unknown	6.4 Not tested Not tested	Not tested Not tested 7.8 ^B^
Mechanism	Clotting	Clotting	Mechanical phenomena during blood collection	Clotting	Pneumatic tube
Treatment (unnecessary)	Kayexalate	None	Unknown	Insulin Bicarbonate Kayexalate	Calcium Bicarbonate Insulin Albuterol

^A^ Tested concurrently. ^B^ K^+^ in plasma component of heparinized whole blood without centrifugation

Prompt diagnosis of pseudohyperkalemia is critical to avoid iatrogenic hypokalemia from treatment (including emergency hemodialysis) for pseudohyperkalemia [5–7, 11]. When significant leukocytosis, thrombocytosis, hyperproteinemia, or hyperlipidemia are present, whole blood potassium measurements should be utilized [13]. In emergent scenarios where potassium levels are critically needed for subsequent decision-making, whole blood testing should also be utilized [13]. Our case indicates that even with lab’s manual flagging system for leukocytosis, pseudohyperkalemia can still occur. This can suddenly happen to the same patient in the same lab on the same day. On suspicion of pseudohyperkalemia in the presence of leukocytosis, a whole blood sample should be walked to the lab, without delay, for chemistry test.

Through this investigation, the lab realized that our manual flagging is not functioning all the time. The main lesson learned from this incident is to automate the lab flagging system, in an attempt to eliminate pseudohyperkalemia associated with leukocytosis, and in a broader range, to prevent other false lab values. The potential benefit is enormous if all hospitals and labs in the world are considered.

In conclusion, pseudohyperkalemia associated with leukocytosis still occurs. This is the first case of pneumatic tube transport causing pseudohyperkalemia associated with AML. When significant leukocytosis, thrombocytosis, hyperproteinemia, or hyperlipidemia is present, whole blood should be utilized for potassium measurements and walked to the lab instead of sent by pneumatic tube transport. Even in a lab with a manual flagging system, there is still room to improve by implementing an automatic flagging system.

## Data Availability

No datasets were generated or analysed during the current study.

## Abbreviations

AML: Acute myeloid leukemia
LT/CLS: Lab technicians/Clinical lab scientists
PTT: Pneumatic tube transport
